# A synthesis of the theories and concepts of early human evolution

**DOI:** 10.1098/rstb.2014.0064

**Published:** 2015-03-05

**Authors:** Mark A. Maslin, Susanne Shultz, Martin H. Trauth

**Affiliations:** 1 Department of Geography, University College London, Pearson Building, Gower Street, London, UK; 2 Faculty of Life Sciences, University of Manchester, Manchester, UK; 3 University of Potsdam, Institute of Earth and Environmental Science, Karl-Liebknecht-Street 24-25, Potsdam 14476, Germany

**Keywords:** human evolution, East Africa, palaeoclimatology, hominin, pulsed climate variability framework

## Abstract

Current evidence suggests that many of the major events in hominin evolution occurred in East Africa. Hence, over the past two decades, there has been intensive work undertaken to understand African palaeoclimate and tectonics in order to put together a coherent picture of how the environment of Africa has varied over the past 10 Myr. A new consensus is emerging that suggests the unusual geology and climate of East Africa created a complex, environmentally very variable setting. This new understanding of East African climate has led to the *pulsed climate variability hypothesis* that suggests the long-term drying trend in East Africa was punctuated by episodes of short alternating periods of extreme humidity and aridity which may have driven hominin speciation, encephalization and dispersals out of Africa. This hypothesis is unique as it provides a conceptual framework within which other evolutionary theories can be examined: first, at macro-scale comparing *phylogenetic gradualism* and *punctuated equilibrium*; second, at a more focused level of human evolution comparing *allopatric speciation*, *aridity hypothesis*, *turnover pulse hypothesis*, *variability selection hypothesis*, *Red Queen hypothesis* and *sympatric speciation* based on sexual selection. It is proposed that each one of these mechanisms may have been acting on hominins during these short periods of climate variability, which then produce a range of different traits that led to the emergence of new species. In the case of *Homo erectus* (*sensu lato*), it is not just brain size that changes but life history (shortened inter-birth intervals, delayed development), body size and dimorphism, shoulder morphology to allow thrown projectiles, adaptation to long-distance running, ecological flexibility and social behaviour. The future of evolutionary research should be to create evidence-based meta-narratives, which encompass multiple mechanisms that select for different traits leading ultimately to speciation.

## 1. Introduction

Human evolution is characterized by speciation, extinction and dispersal events that have been linked to both global and/or regional palaeoclimate records [1–7]. Many theories have been proposed to link environmental changes to these human evolution events [8–11]. This synthesis paper presents each of these theories in the context of the *pulsed climate variability conceptual framework* [11,12], which has been developed from the latest tectonic and palaeoclimate data from East Africa. This greater understanding of the past climate of East Africa suggests that different hominin species or, at the very least, different emerging traits within a species could have evolved through various different mechanisms that are described by the *turnover pulse hypothesis*, *aridity hypothesis*, *variability selection hypothesis, Red Queen hypothesis*, allopatric or sympatric speciation.

## 2. Overview of human evolution

**Figure 1.**
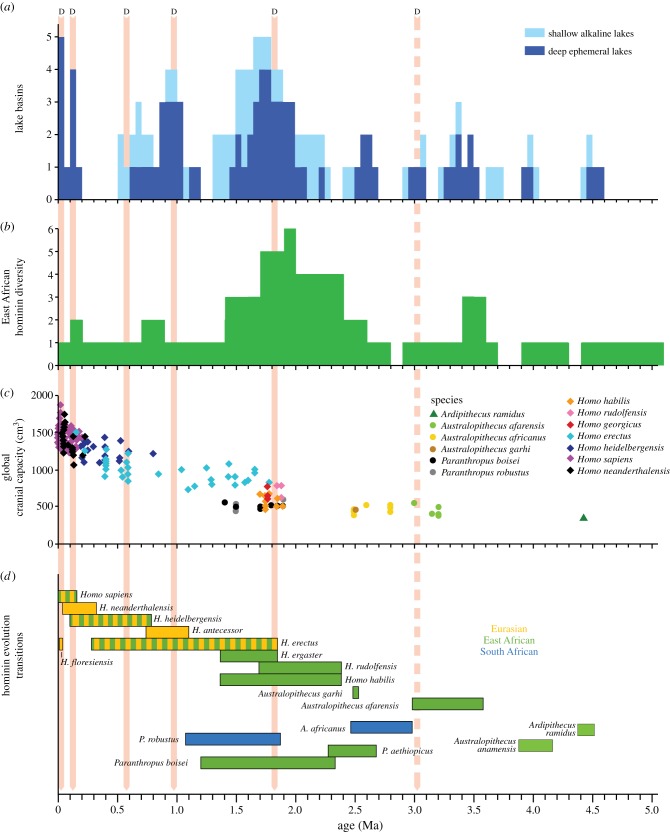
(*a*) The East African Rift Valley lake variability shown as the number of basins containing deep or shallow lakes. Lake basin occupation was calculated by collating the published geological evidence for the appearance of either deep ephemeral or shallow alkaline lakes in seven major basins (see §2). (*b*) East African hominin species diversity over time, which was calculated every 100 kyr interval using first (FAD) and last appearance dates (LAD) from the literature [7]. (*c*) Hominin brain capacity estimates for Africa and for Africa and Eurasia combined. Hominin specimen dates and brain size estimates were taken from Shultz *et al*. [7]. *Homo erectus* and *H. ergaster* were treated as a ‘super-species’ referred to in the figure key as *Homo erectus* (*sensu lato*). (*d*) The age range for key hominin species from Shultz *et al*. [7]. Hominin dispersal dates were estimated by FAD of hominin specimens outside the East African Rift System and are shown by the pink bars labelled ‘D’ (arrows show out of Africa, dotted within Africa only); however, it is stressed that these are liable to large dating errors and can change significantly with new discoveries. (Online version in colour.)

The recent expansion of the hominin fossil record has been dramatic, with 11 new species and four new genera named since 1987. This richer fossil record has provided two major improvements. First, this has led to a much greater understanding of the range of variation in the hominin phenotype, including in ‘real’ biological populations with evidence from Atapuerca, Dmanisi and Hadar. Second, extensive use of new dating techniques has provided chronological precision to link those phenotypes to the environments in which they evolved. However, the fossil record is still very limited with many gaps (figure 1); the most significant for this study is the lack of cranial capacity data between 2 and 2.5 Ma [7]. There is also considerable discussion about defining the new species and genera [13,14], which has an influence on understanding changes in overall hominin diversity. However, conflating or expanding the defined species has little overall influence on the diversity pattern, the pattern of species first appearance dates suggests contemporary speciation events [11]. First appearance dates are dependent on taphonomy and sampling biases; however, the consistency of hominin first appearance dates (FAD) in East Africa supports this region as the primary location of speciation events. The other key debate is where all the new hominin species evolved. The fossil record at the moment suggests that the majority of the new species evolved in East Africa and then dispersed outwards. This is supported by the current brain capacity evidence, which suggests brain expansion occurs first in East Africa and only appears elsewhere once there has been a dispersal event [15]. However, it should be noted that other authors suggest the possibility of South Africa, European and Asian origins for hominin speciation (e.g. [16,17]).

The fossil record suggests four main stages in hominin evolution: (i) the appearance of the earliest (proto) hominins attributed to the genera *Sahelanthropus*, *Orrorin* and *Ardipithecus* between 4 and 7 Ma, (ii) the appearance of the *Australopithecus* genus around 4 Ma and the appearance of the robust *Paranthropus* genus around 2.7 Ma, (iii) the appearance of the genus *Homo* around the Plio-Pleistocene boundary between 1.8 and 2.5 Ma, and (iv) the appearance of *H. heidelbergensis* at 800 ka and anatomically modern humans around 200 ka. The taxonomic classification of many specimens, as well as their role in the evolution of modern humans, is continually discussed (e.g. [13,14]). What is not disputed is that, apart from *Sahelanthropus* remains from Chad, all the earliest specimens for each of the main genera were found in the East African Rift System [18].

The earliest disputed hominin is *Sahelanthropus tchadensis*, dated to approximately 7 Ma [19]. The remains are limited to cranial fragments that suggest a mosaic of hominin and non-hominin features and a brain size equivalent to modern chimpanzees [20]. The lack of post-cranial remains makes it extremely difficult to reconstruct its lifestyle and whether it was bipedal or whether it was truly a hominin. The next putative hominin is *Orrorin tungenesis* from Western Kenyan deposits aged around 6 Ma [21], but its taxonomic position, lifestyle and locomotion are all disputed owing to the fragmentary nature of the specimens. Both *Sahelanthropus* and *Orrorin* have been suggested to be members of a clade that includes *Ardipithecus* [20]. The oldest member of the *Ardipithecus* genus is *A. kadabba*, whose fossil evidence consists only of fragmentary teeth and skeletal remains dated to approximately 5.5 Ma [22]. A much more extensive fossil record exists for the second member of the genus, *A. ramidus. Ardipithecus* had brain and body sizes roughly equivalent to modern chimpanzees, their teeth indicate a highly omnivorous diet and their post-crania suggest a lifestyle of arboreality coupled with primitive bipedality [23]. The fauna and vegetation associated with the *A. ramidus* specimens in the Awash Valley, Ethiopia, dating to around 4.4 Ma suggest woodland–forest matrix habitats, associated with significant rainfall and water availability [23–25]. This appearance of bipedality in closed woodland environments undermines theories of bipedality evolving exclusively as an adaptation to open habitats.

The first members of the *Australopithecus* genus, attributed to *A. anamensis*, appeared around 4 Ma [26]. These individuals show strong evidence of bipedality combined with primitive cranial features. They are followed by *A. afarensis*, which is very well known from the fossil record and includes the remarkably complete ‘Lucy’ specimen. *Afarensis* still retains a small brain size, yet the post-cranial morphology is more similar to modern humans than to apes and suggests a lifestyle strongly adapted to long-distance walking [27]. *Australopithecus africanus*, the first hominin found in South Africa, is similar to *A. afarensis* but with more ape-like limb proportions yet less primitive teeth [28]. The longer femur in *A. afarensis* as compared with *A. africanus* suggests longer strides and a more efficient walking style [28]. The final gracile australopith is *A. garhi*, associated with 2.5 Ma old deposits in the Awash Valley [29]. In a separate development, a group of hominins with robust dentition and jaw muscles appeared around 2.5 Ma. These hominins, generally attributed to the *Paranthropus* genus, include the East African *P. aethiopicus* (2.5 Ma) and *P. boisei* (2.3–1.2 Ma), and the Southern African *P. robustus* (1.8–1.2 Ma). These species have been attributed to more open habitats [25], though the evidence to support this inference has been questioned [30].

The earliest fossil evidence of *Homo* comes from 1.8 to 1.9 Myr old deposits in the East African Rift Valley. *H. habilis* had a gracile morphology similar to the australopithecines [18], and a brain size only slightly larger, leading to some arguing it should not be classified as *Homo* [31]. *H. habilis* was then followed by the appearance of *H. erectus sensu lato*, which is associated with sweeping changes in brain size, life history, and body size and shape. Post-cranially, *H. erectus* is very similar to anatomically modern humans. Inferences from fossil demography are that development slowed down, coupled with decreased inter-birth intervals. The final stages in the evolution of modern humans were the appearance of *H. heidelbergensis* around 800 ka and anatomically modern humans around 200 ka.

Arguably, the most important episode in hominin evolution occurred in East Africa around 1.8–1.9 Ma when hominin diversity reached its highest level with the appearance of the robust *Paranthropus* species, as well as the first specimens attributed to genus *Homo* (*sensu stricto*). In addition to speciation, a second major process that began during this period was the episodic migration of hominins out of the Rift Valley and into Eurasia. This period also witnessed the most dramatic increases in hominin brain size; early representatives of the *H. erectus sensu lato* (*H. erectus* and *H. ergaster*) in Africa had a brain that was more than 80% larger than the gracile australopithecine *A. afarensis* and approximately 40% larger than *Homo (Australopithecus) habilis* (figure 1). By contrast, from the appearance of the early australopithecines until the appearance of the first member of the genus *Homo,* there was remarkably little change in hominin brain size.

The emergence of the *H. erectus sensu lato* in East Africa represents a fundamental turning point in hominin evolution. The dramatic increase in brain size was also accompanied by changes in life history (shortened inter-birth intervals, delayed development), pelvic morphology (see [32,33] in this issue), body size and dimorphism, shoulder morphology allowing throwing of projectiles [34], adaptation to long-distance running [35], ecological flexibility [36] and social behaviour [37]. Some of these changes are consistent with a change in strategy towards flexibility and the ability to colonize novel environments. By contrast, the robust *Australopithecus* sp. adopted specialized habitat and dietary strategies [38,39]. Thus, two strategies arose during this period, one of increased flexibility and one of increased specialization. With the appearance of *H. erectus*, brain size increased significantly and continued to increase over the following 500 kyr, followed by additional step increases between 0.8 and 1 Ma, at 200 ka, and finally again at 100 ka ([7], figure 5). These final stages of increased brain capacity were due to the appearance of *H. heidelbergensis* around 800 ka, *H. denisovan* around 600 ka, *H. neanderthal* around 300 ka and anatomically modern humans around 200 ka.

## 3. Theories of early human evolution

Environmental pressures have long been assumed to play a key role in hominin speciation and adaptation [40] and a number of iconic theories have been developed to frame and develop the discussion of hominin evolution. Table 1 tries to put these key theories into the context of overarching evolutionary theory. Although the split between *phylogenetic gradualism* and *punctuated equilibrium* is artificial, it does provide a starting point with which to discuss theories of early human evolution. In table 1, gradualism has been split into constant and variable evolution rates to reflect the full range of current opinions.

**Table 1.** Early human evolutionary theories placed in the context of overall evolutionary theory and modes of climatic change.

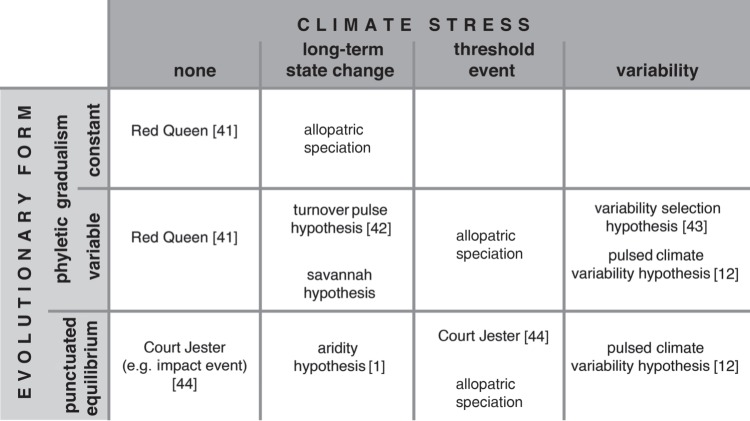

The first key environmental theory to explain bipedalism was the *savannah hypothesis*, which suggested that hominins were forced to descend from the trees and adapted to life on the savannah facilitated by walking erect on two feet. This theory was refined as the *aridity hypothesis*, which suggested that the long-term trend towards increased aridity and the expansion of the savannah was a major driver of hominin evolution [1,38,45]. A key addition to this theory was the suggestion that during periods when aridification accelerated, owing to thresholds in the global climate system, then thresholds in evolution were reached and major hominin speciation events occurred [1].

The *turnover pulse hypothesis* [42,46–48] was originally developed to explain discrete patterns in ungulate speciation, and suggests that acute climate shifts drove adaptation and speciation. Vrba [46] recognized that environmentally induced extinctions hurt specialist species more than generalist species. Hence, when there is an environmental disruption, the generalists will tend to thrive by using new environmental opportunities and by moving elsewhere to take advantage of other areas that have lost specialist species. The specialists will experience more extinctions, and therefore an increased speciation rate within their group. This would lead to more rapid evolution in isolated areas, i.e. allopatric speciation, whereas the generalists will become more spread out.

The *variability selection hypothesis* advocates the role of environmental unpredictability in selecting for behavioural or ecological flexibility [10,43,49–51]. This theory develops the original *turnover pulse hypothesis* but instead splits species into their varying ability to adapt and evolve to a more variable and unpredictable environment. The *variability selection hypothesis* emphasizes the long-term trends toward a drier and more variable climate. It, however, struggles to explain the current palaeoanthropological evidence that suggests a pulsed/threshold nature of hominin speciation and migration events.

More recently, it has been suggested that periods of climate stability may be equally important in driving human evolution, dispersal and technological innovation (e.g. [15,52,53]). Relatively long periods of climate stability could invoke the *Red Queen hypothesis* or *sympatric evolution* owing to sexual selection. The *Red Queen hypothesis* suggests that continued adaptation is needed in order for a species to maintain its relative fitness among co-evolving systems [54] and that biotic interactions, rather than climate, are driving evolutionary forces. It is based on the Red Queen's race in Lewis Carroll's *Through the Looking-Glass*, when the Queen says; ‘It takes all the running you can do, to keep in the same place’ [44]. However, for this to occur, it is reasonable to assume that a relatively highly productive environment has to exist so that competition rather than resources is the dominant control. At Koobi Fora (Northern Kenya) there is evidence for multiple hominin species, including *P. boisei, H. erectus* spp., *H. habilis* and *H. rudolfensis* attributed to the period of maximal lake coverage (approx. 1.8–1.9 Ma), and hence the highest availability of resources; it might be postulated that these hominins evolved as a result of competition with each other and other animals.

**Figure 2.**
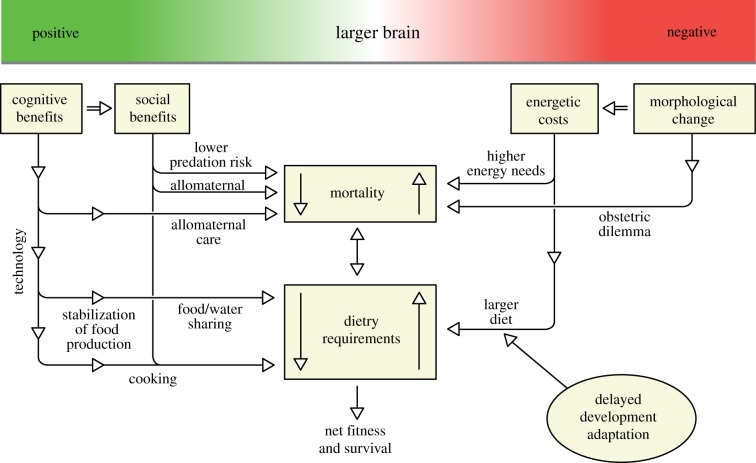
Expensive brain hypothesis. (Online version in colour.)

It may also be possible that certain traits, such as a large brain, became a key characteristic in sexual selection that hence drove sympatric evolution. The *social brain hypothesis* [55,56] suggests that enhanced cognitive ability would provide the ability to strongly influence groups or tribes of hominins and hence control the distribution of resources. It would also help social cohesion and thus the ability of individuals to ensure allomaternal care, reducing the effects of the obstetric dilemma (see [57] in this issue). The *social brain hypothesis* could apply equal well to periods of high or low resources as it can be seen as an internal arms race to develop great cognitive skills to enable greater social control. In additional to this is the *expensive brain framework* [56,58] which tries to understand the advantages of having enhanced cognitive ability in terms of food production and sharing, predation reduction and allomaternal care compared with the negative impacts such as increase food requirement and increased infant and mother mortality (figure 2). Both of these theories provide an essential link between the mechanisms driving evolution and the biological response.

Finally, a direct development of the *variability selection hypothesis,* which incorporates the latest palaeoenvironmental reconstructions and the role of both stability and instability is the *pulsed climate variability framework*, which highlights the role of short periods of extreme climate variability specific to East Africa in driving hominin evolution [12]. This framework is discussed in §4 along with how the other evolutionary theories may be applied given the new environmental context.

## 4. Pulsed climate variability conceptual framework

Over the past two decades, intense work on African palaeoclimate and tectonics has allowed us to begin to put together a coherent picture of how the environments of eastern and southern Africa have changed over the past 10 Myr. Tectonics has altered the landscape of East Africa dramatically over this period of time. It changed from a relatively flat, homogenous region covered with tropical mixed forest, to a heterogeneous region, with mountains over 4 km high and vegetation ranging from desert to cloud forest. Tectonic events such as these are associated with a variety of biotic changes. Over the Oligocene and Miocene progressive uplift of East Africa split the pan-Africa rainforest which joined the Congo with East Africa resulting in endemic species in East Africa emerging at 33, 16 and 8 Ma [59]. During the Plio-Pleistocene, there is evidence from soil carbonates [60–63], marine sediment *n*-alkane carbon isotopes [64–66] and fossilized mammal teeth [67,68] that there was a progressive vegetation shift from C_3_ plants to C_4_ plants during the Pliocene and Pleistocene. This vegetation shift has been ascribed to increased aridity owing to the progressive rifting and tectonic uplift of East Africa [45]. The aridity trend is also supported by a number of climate model simulations [69–71]. These studies demonstrate that as uplift increases, wind patterns become less zonal resulting in a decrease in regional rainfall. Hence as elevation increases, a rain shadow effect occurs that reduces moisture availability on the Rift Valley mountain side, producing the strong aridification trend evident in palaeoenvironmental records [69,70].

In addition to contributing towards the aridification of East Africa, the tectonic activity also produced numerous basins suitable for lake formation [72]. The southward propagation of rifting, including the formation of faults and magmatic activity, is also reflected in the earliest formation of lake basins in the northern parts of the rift. For example, the Middle and Upper Miocene saw the beginning of lakes in the Afar, Omo-Turkana and Baringo-Bogoria Basins, but the oldest lacustrine sequences in the central and southern segments of the rift in Kenya and Tanzania are of Early Pliocene age [73,74]. Palaeo-lakes in the northern part of the East African Rift Valley thus formed earlier than in the south. However, if tectonics were the sole control over the appearance and disappearance of lakes, then either a north–south or north-west–south-east temporal pattern would be expected. By contrast, what is observed is the synchronous appearance of large deep lakes across a large geographical area at specific times [2], suggesting a regional climatic control. Moreover, there is growing evidence for significant late Cenozoic lake periods between 4.6–4.4 Ma, 4.0–3.9 Ma, 3.6–3.3 Ma, 3.1–2.9 Ma, 2.7–2.5 Ma, 2.0–1.7 Ma, 1.1–0.9 Ma and 0.2–0 Ma before present in East Africa [2,11,74]. These occurrences correlate with the 400- and 800-kyr components of the eccentricity cycle, suggesting a major role in lake formation for extreme amplitude fluctuations in precession (figure 4). During each of the lake phases there is evidence that the lakes appear and disappear rapidly in time with precessional forcing [1,45,75–86]. Deino *et al*. [77] and Kingston *et al*. [78] have found that the major lacustrine episode of the Baringo Basin in the Central Kenyan Rift between 2.7 and 2.55 Ma actually consisted of five palaeo-lake phases separated by a precessional cyclicity of approximately 23 kyr, while Magill *et al*. [84] have found biomarker stable carbon isotope evidence in Olduvai lake sediment of precessional forced variations between open C_4_ grasslands and C_3_ forest between 1.8 and 1.9 Ma. There is also evidence for precessional forcing of the 1.9–1.7 Ma lake phase indentified in the KBS Member of the Koobi Fora Formation in the northeast Turkana Basin in Kenya [80,87]. During the same period, an oxygen isotope record from the Buffalo Cave flowstone (Makapansgat Valley, Limpopo Province, South Africa) shows clear evidence of precessionally forced changes in rainfall [79]. The occurrences of these environmental changes are in phase with increased freshwater discharge and thus sapropel formation in the Mediterranean Sea [88–90] and coincide with dust transport minima recorded in sediments from the Arabian Sea [1,45,91]. Hence, the lake records from East Africa and the Arabian Sea dust records document extreme climate variability with precessionally forced wet and dry phases.

**Figure 3.**
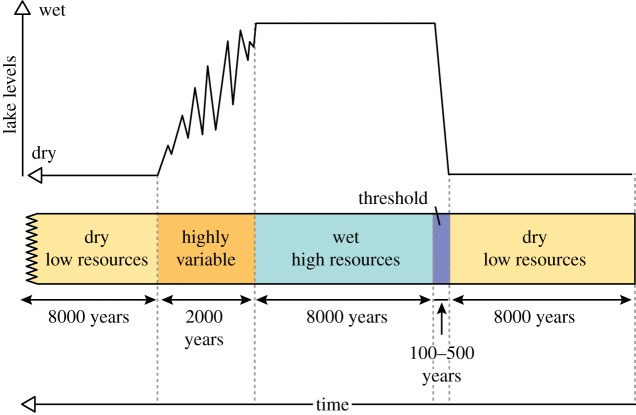
Illustration of the lake variations in East Africa during periods of extreme climate variability [11,86]. Usually there are four or five of these cycles during any particular extreme period. (Online version in colour.)

**Figure 4.**
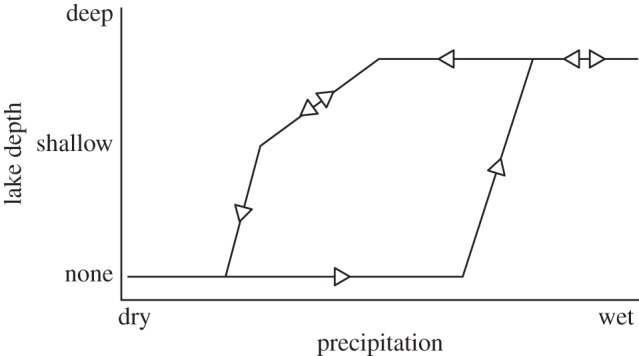
Bifurcated relationship between changing regional precipitation and lake depth in the East African Rift Valley.

In summary, the *pulsed climate variability framework* suggests there are periods of extreme climate variability every 400 or 800 kyr driven by the eccentricity maxima when lakes rapidly grow and fill much of the Rift Valley and then rapidly disappear. Wilson *et al*. [86], using evidence from Pliocene diatomite deposits in the Baringo Basin, suggest that the lakes appear rapidly, remain part of the landscape for thousands of years, then disappear in a highly variable and erratic way [77,82]. In fact, the absence of shallow-water (littoral) diatom species at key Plio-Pleistocene lake deposits [78,82,86] suggests that the lakes appeared in only a few hundred years. Figure 3 shows a compilation of what a generic extreme wet–dry cycle may have looked like with a threshold at the beginning of the wet phase and a prolong highly variable period at the end of the wet phase. There would be four or five of these cycles during each of the periods of extreme climate variability. The different appearance and disappearance of the lakes is also consistent with the idea of a bifurcated relationship between climate and lake presence [92]. Figure 4 shows that precipitation needs to increase significantly before lake growth can initiate, but once it has there are some key feedbacks which accelerate the expansion of the lakes. The most important is the change in the local lapse rate owing to increased moisture in the atmosphere. Hence the increased local relative humidity reduces the evaporation–precipitation balance, increasing the moisture in the atmosphere. When lake become more established the increased moisture changes the vegetation and more bushes and trees appear which subsequently increase the evapo-transpiration, further increasing the moisture in the atmosphere. These same feedbacks also resist the drying out of the lake when precessionally driven rainfall starts to reduce. This leads to a period of up to 2 kyr when the lake expands and contracts finally before there is not enough moisture in the region to sustain any sort of lake. Recent evidence of this lake behaviour has been found using radiocarbon dating of the palaeo-Lake Suguta in Northern Kenya [93,94].

It should be stressed, however, that the *pulsed climate variability framework* only applies up to about 800 ka. After this time, the Early–Middle Pleistocene transition (which was previously known as the Mid-Pleistocene Transition or Revolution [95]) occurs which marks a prolongation and intensification of glacial–interglacial climate cycles, which have an increasing influence on tropical climates. Hence post 800 ka, the climate of the tropics becomes more complicated and fragmented as it is influenced both by localized influences of orbital forcing as well as the evermore global influence of the glacial–interglacial cycles [11].

## 5. Human evolution theories within the new framework

**Figure 5.**
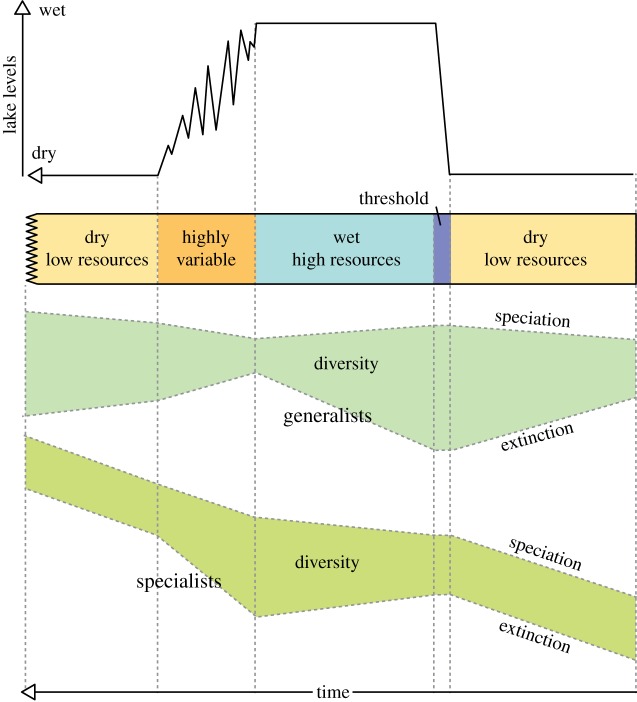
An interpretation of how the *turnover pulse hypothesis* could be placed within the *pulsed climate variability framework*. (Online version in colour.)

**Figure 6.**
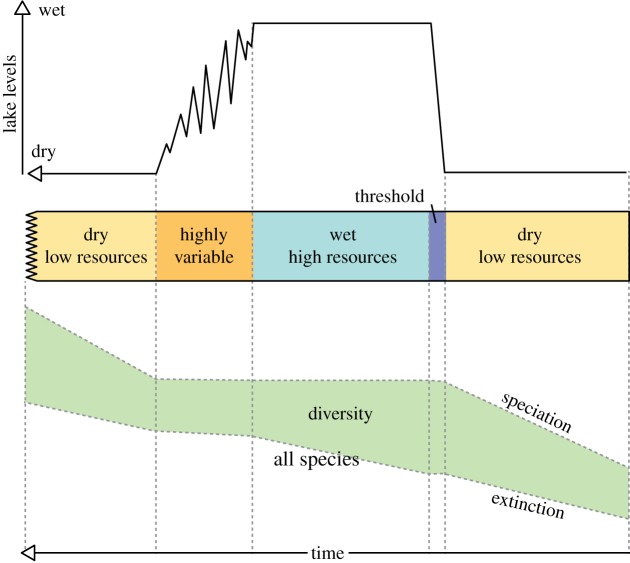
An interpretation of how the *aridity hypothesis* could be placed within the *pulsed climate variability framework*. (Online version in colour.)

**Figure 7.**
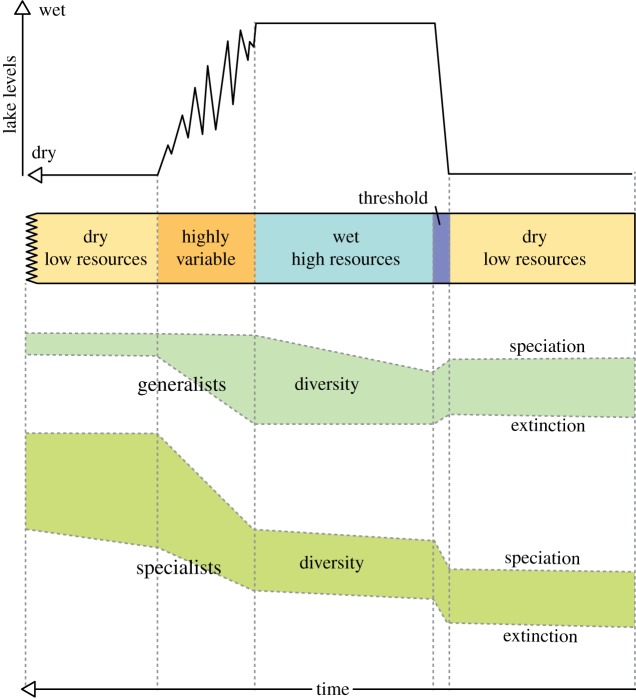
An interpretation of how the *variability selection hypothesis* could be placed within the *pulsed climate variability framework*. (Online version in colour.)

**Figure 8.**
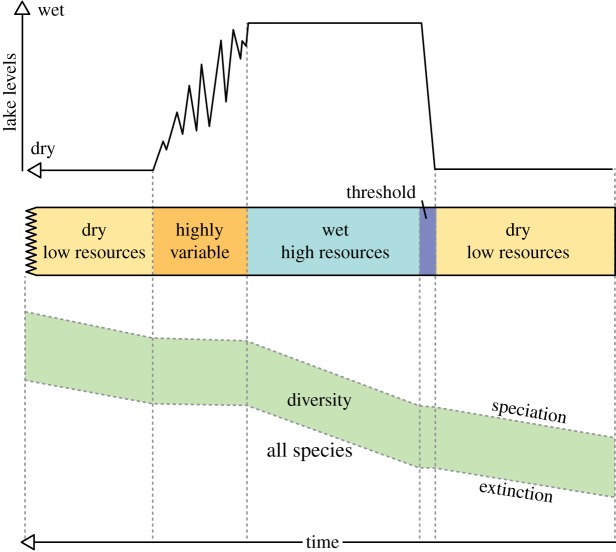
An interpretation of how the *Red Queen hypothesis* could be placed within the *pulsed climate variability framework*. (Online version in colour.)

**Figure 9.**
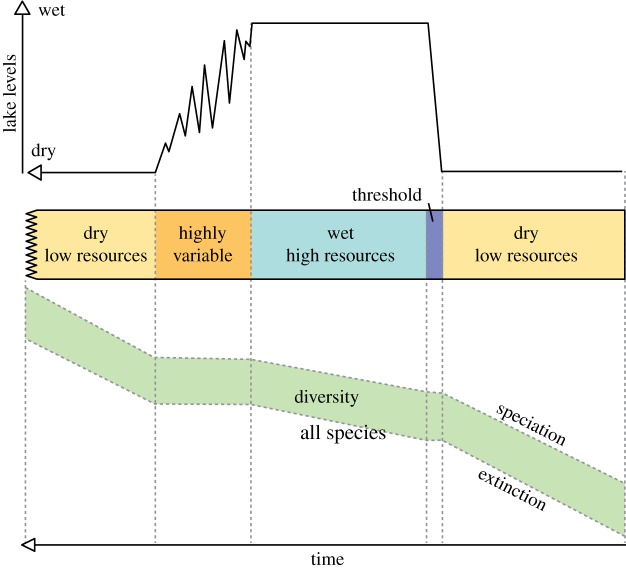
An interpretation of how the *allopatric speciation hypothesis* could be placed within the *pulsed climate variability framework*. (Online version in colour.)

Figures 5–9 illustrate how the *pulsed climate variability framework* helps to conceptualize the different theories of early human evolution. Figure 5 shows how the *turnover pulse hypothesis* would operate through one of these extreme climate cycles. Vrba [46] suggested that environmental changes would affect specialist and generalist species differently. During dry periods, the extinction rates of generalist species would reduce as they would be better able to find resources, while specialist species would struggle having lost their environmental niche and their competitive advantage (figure 5). Speciation would be much higher in the specialist species during dry periods as they try to adapt to the new habitats. By contrast, during the wet periods and to a lesser extent the high variable periods generalist species would suffer as specialists would have a lot more niches to fill and thus would outcompete the generalists. Figure 6 illustrates possible changes that could have occurred owing to the *aridity hypothesis* [1,45], which suggest that speciation mainly occurs during periods of dryness with low resources. Figure 7 illustrates the *variability selection hypothesis* [10,43,50,51] that develops the original *turnover pulse hypothesis* but instead splits species into their varying ability to adapt and evolve to a more variable and unpredictable environment. Hence, generalists undergo more extinction and specialists more speciation during the highly variable climate period in between the long wet and arid phases. Figure 8 illustrates the *Red Queen hypothesis* that suggests continued adaptation is needed in order to keep up with other species which are also evolving. Figure 8 assumes that a relatively high-energy environment would provide more resources and therefore more energy in the system to enable inter-species competition. The structure of figure 8 could also apply to *sympatric evolution* owing to sexual selection. Finally, figure 9 illustrates *allopatric evolution* that suggest geographically isolated populations can evolve independently. In the Rift Valley during the extreme dry periods north–south and east–west migration was very difficult so it would have created isolated populations. The same is true of extreme wet periods because when the lakes completely fill the rift basins north–south and east–west migration would be again difficult, creating isolated populations. Only during the highly variable period and the threshold change would it be possible easily to move up and down and across the Rift Valley. Recent evidence from Wilson *et al*. [86] suggests there were millennial-scale fluctuations in lake level during the extreme wet periods; hence movement between populations may have been possible during the wet phases, limiting the isolation.

Figures 5–9 are just our interpretations of how these major theories of human evolution could be placed within the *pulsed climate variability framework.* We would encourage colleagues to use this more visual approach to provide their own interpretation of how changing environments would interact with different theories of early human evolution.

## 6. Conclusion

The *pulsed climate variability framework* therefore takes the latest palaeoclimate understanding of East Africa and provides a framework within which to understand the causes of early human evolution. Different species or, at the very least, different emerging traits within a species could have evolved through various mechanisms including the *turnover pulse hypothesis*, *aridity hypothesis*, *variability selection hypothesis* or allopatric speciation. This is exemplified by the case of *H. erectus* (*sensu lato*) which exhibits changes in life history (shortened inter-birth intervals, delayed development), pelvic morphology, body size and dimorphism, a shoulder morphology that enables projectile use, adaptation to long-distance running, ecological flexibility, social behaviour which may have included cooking. Each one of these traits could have been forced by a different evolutionary mechanism operating at a different part of the environmental cycle.
