# Correction to ‘A synthesis of the theories and concepts of early human evolution’

**DOI:** 10.1098/rstb.2020.0344

**Published:** 2020-09-21

**Authors:** Mark A. Maslin, Susanne Shultz, Martin H. Trauth

*Phil. Trans. R. Soc. B* **370**, 20140064. (Published online 5 March 2015). (doi:10.1098/rstb.2014.0064)

*Text Recycling Correction Notice*: This article contains text that has been previously published by the author. The purpose of this correction is to amend the publication record by clarifying where overlap occurs in this article. Parts of §§2, 3 and 4 of this article also appear in Maslin *et al.* (2014) ‘East African climate pulses and early human evolution’ ([11] in the article). Figure 1*a*–*c* was taken from Shultz & Maslin (2013) ‘Early human speciation, brain expansion and dispersal influenced by African climate pulses' [15].

